# Motor Control Adherence to the Two-thirds Power Law Differs in Autistic Development

**DOI:** 10.1007/s10803-024-06240-6

**Published:** 2024-01-27

**Authors:** Emily Fourie, Szu-Ching Lu, Jonathan Delafield-Butt, Susan M. Rivera

**Affiliations:** 1 Department of Psychology, University of California, Davis, Davis, CA USA; 2 Center for Mind and Brain, University of California, Davis, Davis, CA USA; 3 Laboratory for Innovation in Autism, University of Strathclyde, Glasgow, Scotland, UK; 4 Strathclyde Institute of Education, University of Strathclyde, Glasgow, Scotland, UK; 5 College of Behavioral and Social Sciences, University of Maryland, College Park, MD USA

**Keywords:** Autism, Motor, Movement, Two-thirds Power law, Jerk

## Abstract

Autistic individuals often exhibit motor atypicalities, which may relate to difficulties in social communication. This study utilized a smart tablet activity to computationally characterize motor control by testing adherence to the two-thirds power law (2/3 PL), which captures a systematic covariation between velocity and curvature in motor execution and governs many forms of human movement. Children aged 4–8 years old participated in this study, including 24 autistic children and 33 typically developing children. Participants drew and traced ellipses on an iPad. We extracted data from finger movements on the screen, and computed adherence to the 2/3 PL and other kinematic metrics. Measures of cognitive and motor functioning were also collected. In comparison to the typically developing group, the autistic group demonstrated greater velocity modulation between curved and straight sections of movement, increased levels of acceleration and jerk, and greater intra- and inter-individual variability across several kinematic variables. Further, significant motor control development was observed in typically developing children, but not in those with autism. This study is the first to examine motor control adherence to the 2/3 PL in autistic children, revealing overall diminished motor control. Less smooth, more varied movement and an indication of developmental stasis in autistic children were observed. This study offers a novel tool for computational characterization of the autism motor signature in children’s development, demonstrating how smart tablet technology enables accessible assessment of children’s motor performance in an objective, quantifiable and scalable manner.

## Introduction

Motor differences in autism (ASD) have been clearly identified (Trevarthen & Delafield-Butt, 2013). In a recent study of nearly 12,000 children with ASD between 5 and 15 years of age, 87% of children were at risk for motor impairment (Bhat, 2020). Other research has focused on characterizing movement atypicalities early in development. Studies using wearable sensors have detected decreased acceleration in spontaneous and directed limb movements of high-risk infants (Wilson et al., 2021) and toddlers (Focaroli et al., 2016), compared to low-risk and non-autistic counterparts. Other studies employing motion tracking systems have observed that toddlers with ASD show decreased prospective motor control (i.e., performed fewer predictive reaches) during a ballcatching task (Ekberg et al., 2016), as well as differential movement kinematics during free play (Yang et al., 2019). The growing use of technology-based assessment, as demonstrated in these studies, has revealed that atypical motor movement is present within the first 3 years of life, before the normative age of diagnosis.

These trends in motor impairment continue throughout development, especially as fine motor skills become increasingly important in daily activities. Studies assessing handwriting and drawing have revealed larger peak velocity, less smooth movements, and more variable size trajectories, and a correlation between writing performance and ASD severity (Beversdorf et al., 2001; Grace et al., 2017). In executing upper limb reach-to-grasp movements, those with ASD have been shown to take longer to prepare and execute movements, show greater levels of jerk (rate of acceleration), and more movement units (phases of acceleration with deceleration) than their counterparts (Cook et al., 2013; Torres et al., 2013; Yang et al., 2014). Precision gripping experiments have revealed that children with ASD show increased variability, implicating differences in feedback control and motor planning (Mosconi et al., 2015; Wang et al., 2015). Distinctive kinematic profiles in individuals with ASD have emerged in other motor movements, such as ball throwing (Staples & Reid, 2010), gait (Calhoun et al., 2011; Cho et al., 2022; Nobile et al., 2011; Rinehart et al., 2006a; Rinehart et al., 2006a, b), and gameplay with blocks (Ferrara et al., 2016a) and iPads (Anzulewicz et al., 2016; Chua et al., 2021; Lu et al., 2022).

These differences in both fine and gross motor skills may contribute to disruption of more complex forms of socially embedded movement. In studies designed to elicit gestures both through command or imitation, autistic children make more orientation and distortion errors (Dewey et al., 2007; Gordon & Watson, 2015), and demonstrate poorer overall gesture performance, marked by atypical hand posture (Fourie et al., 2020). Additionally, individuals with ASD show impairments in their ability to imitate others’ actions (Dewey et al., 2007; Williams et al., 2004). Research also reveals differences in movement during social interactions: those with ASD demonstrate lower social motor synchronization with a counterpart (Fitzpatrick et al., 2017), as well as excessive and less complex movement in face-to-face conversation (Zhao et al., 2021). These studies make clear the larger role that motor atypicality may play in nonverbal communication and social interaction, core areas of concern in ASD. Some have even theorized that motor atypicalities, especially in the area of prospective motor control, represent a primary manifestation or “intermediate phenotype” of ASD, which subsequently leads to difficulties in the social domain (Casartelli et al., 2016; Trevarthen & Delafield-Butt, 2013).

Despite the growing body of evidence demonstrating that motor impairments are prevalent and pervasive in ASD, motor features are not a part of the autism diagnostic criteria. The DSM-5 (American Psychiatric Association, 2013), currently used to diagnose autism, includes repetitive and stereotyped motor movements (RRBs), but not coordination difficulties in general motor movement. As such, motor impairments are largely underdiagnosed and undertreated in ASD (Bhat, 2020). Recently, however, a number of researchers have made a case for including motor impairment in the diagnostic process (Bhat, 2021; Bondioli et al., 2021; Iverson et al., 2019; Licari et al., 2021; Mosconi & Sweeney, 2015), with some work providing support that adding motor domains may better capture heterogeneity (Harrison et al., 2021). However, this perspective has also been challenged, with claims that current motor measures have not been validated in ASD and that poor performance on motor tasks may indicate disinterest rather than primary impairment (Bishop et al., 2022; Crippa, 2022). Yet, given that motor impairment is often the first observable sign of autism, it could potentially serve as a marker for early identification and referral for diagnosis.

Further, motor development is crucial in driving other cognitive processes (Trevarthen & Delafield-Butt, 2017, 2013; Von Hofsten, 2007). Previous studies have highlighted the broad impact that motor impairments can have on development of social and communicative abilities, including both expressive and receptive language development (LeBarton & Landa, 2019; Patterson et al., 2021), as well as empathy and face processing abilities (Casartelli et al., 2016; Cummins et al., 2007; Gallese et al., 2013; Iverson, 2010). Furthermore, difficulties with motor skills can result in challenges with activities of daily living, overall health and independence. As such, early intervention in motor delays could alter the developmental trajectory, improving outcomes in other social cognitive domains, making it a critical area for further investigation.

## The Two-Thirds Power Law

One approach to characterize motor development is through the two-thirds power law (2/3 PL), a kinematic profile which describes a stable covariation between velocity and curvature of movement. The name of the law derives from the exponent in the equation by which it was originally defined:

1 $$Angular{\text{ }}velocity{\text{ }} = {\text{ }}K{\text{ }}*{\text{ }}Curvatur{e^{2/3}}$$

More commonly and as will be used in this study, the relationship is expressed using an alternative, mathematically equivalent equation, based on different variables (and thus resulting in a different exponent):

2 $$Tangential{\text{ }}velocity{\text{ }} = {\text{ }}K{\text{ }}*{\text{ }}Radius{\text{ }}of{\text{ }}curvatur{e^{1/3}}$$

In both equations, K represents a velocity gain factor that remains constant across the movement.

The law defines an inverse relationship between tangential velocity and curvature (the exponent in Eq. 2 is positive because the variable ‘radius of the curvature’ is the *inverse* of ‘curvature’) such that the velocity is lower in more curved parts than in less curved (straighter) parts of the movement. This model captures the tendency of the motor system to optimize movement by maximizing its smoothness. Thus, movement adhering to the law is perceived as being constant and uniform despite having variable velocity (Levit-Binnun et al., 2006; Salomon et al., 2016; Viviani & Stucchi, 1992), is judged to be “natural” even when presented without the context of a human form (Bidet-Ildeil et al., 2006; Salomon et al., 2016), and is more accurately predicted than motion incompatible with the law, both in single dot presentations (Flach et al., 2004) and more ecologically relevant displays of cursive handwriting (Kandel et al., 2000). Further, infants as young as 4 days appear to discriminate between 2/3 PL and constant motion profiles (Méary et al., 2007). These studies suggest that this fundamental characteristic of the motor system strongly influences how we perceive motion, via innate sensitivity to its “biological” smoothness.

This kinematic property of motion extends to several types of movements, including arm and foot trajectories (Ivanenko et al., 2002; Richardson & Flash, 2002), eye movements (De’Sperati & Viviani, 1997), drawing (Viviani & Schneider, 1991), and even movement planning (Viviani & Flash, 1995). Adherence to the law exists independently of the rate of movement, size of the shape, and type of curvilinear path (ellipse, Lissajous curve, cloverleaf; Hicheur et al., 2005; Levit-Binnun et al., 2006; Viviani & Flash, 1995). This attribute is believed to be a key feature which sets biological/human motion apart from most artificially-generated motions (Kandel et al., 2000).

Production of motion compliant with this law is present early in development. Research examining the organization and structuring of spontaneous arm movement in 3- to 5-day-old neonates demonstrated a precise coupling of velocity and curvature (von Hofsten & Rönnqvist, 1993). This property of motor behavior has also been demonstrated in reaching movements of 5- to 9-month old infants, despite the apparent lack of coordination of these movements, and independent of whether a reaching movement resulted in a successful grasp (Fetters & Todd, 1987). These studies provide evidence of an innate tendency to execute movement in line with this law, highlighting the fundamental nature of this property of the motor system. It appears to be an invariant characteristic which requires no skill or practice, and is present across a range of functional and spontaneous movements.

As children begin to develop greater motor control, this velocity-curvature association is also present in their drawing movements, such as the smooth-inertial sections of 2-year-olds’ circular scribbles (Adi-Japha et al., 1998). Two studies examined the law developmentally in samples of 5- to 12-year-old children and found a close coupling of velocity and curvature in both free-hand-drawn (Sciaky et al., 1987) and template-traced ellipses (Viviani & Schneider, 1991). Interestingly, both studies demonstrated that adherence to this law of movement progresses developmentally: with increasing age, there was an increase in the strength of the association between curvature and velocity (Sciaky et al., 1987) as well an increase in the beta value (i.e., the exponent in Eq. 2) toward the adult value of 1/3 (Viviani & Schneider, 1991). In sum, these studies point to the presence of this fundamental property of human motor behavior early in development, with progressive strengthening and tuning through experience over a period of several years.

## Neural Mechanisms Underlying the Power Law

Simple dot motion following the 2/3 PL appears to elicit fMRI activity that is stronger and more widespread than other types of motion (Casile et al., 2010; Dayan et al., 2007). Similar work has been replicated using EEG methodology, in which event-related desynchronization (ERD), considered to reflect cortical motor activity, was stronger and arose faster during observation of motion following the 2/3 PL compared to other motion profiles (Meirovitch et al., 2015). This selectivity and heightened sensitivity indicate that the brain’s perceptual system is tuned to kinematics adhering to this law. The observed pattern of neural activity involves networks associated with motor planning and production, suggesting that our brains evaluate dynamic visual input in relation to our internal motor representations, which center around adherence to the law.

Other research has suggested that this kinematic law may stem from neural coding principles in both perceptual and motor processes (Levit-Binnun et al., 2006). For example, several neurophysiological studies recorded activity from single cells in motor cortex as monkeys performed reaching and drawing movements (Schwartz & Moran, 1999, 2000). The 2/3 PL was evident in the neural correlates of monkey hand movement, and the kinematic components of velocity and directionality could be predicted by firing properties. The authors concluded that execution of movement is constrained by neural processing: the capacity of the system to transmit directional information is limited such that as direction of movement changes around a curved trajectory, the arm slows to reflect these neural constraints. While the precise nature of the mechanism is not yet clear, these studies point to some underlying processes that are specialized for motion adhering to the 2/3 PL.

Taken together, the 2/3 PL of motion is suggested to be subserved by biological, evolutionary and neural mechanisms. It appears to be fundamentally embedded in the motor production system and evident early in development. Thus, this law provides a useful tool for studying motor development in a condition like ASD with early emerging developmental differences. Given its age-related developmental trajectory, the law provides a systematic way to assess developing motor control, across an age when more advanced fine motor skills, like handwriting and picture drawing, emerge. It offers a mathematically defined model which captures a fundamental, biological quality of human movement. The proposed study seeks to capitalize on the nature of this law to investigate motor performance in ASD.

## The Current Study

The current study addressed the question of whether autistic individuals differ from typically developing (TD) individuals in their movement execution. By measuring adherence to the 2/3 PL, we aimed to systemically assess the biological quality of movement that can be quantified according to this model. Research on motor kinematics in ASD has typically examined standard measures of velocity and acceleration and has revealed discrepant findings. For example, some studies report slower peak velocity (Glazebrook et al., 2016) while others show faster velocity (Forti et al., 2011; Grace et al., 2017) of autistic movement compared to TD, depending on the paradigm, task objectives and context (Lu et al., 2022).

No studies have investigated adherence of movement to the 2/3 PL in ASD, which may be a better metric given that this law reflects motor control and governs execution of many forms of human movement.^1^ Studies which do examine a closely-related metric, jerk, appear to show consistent findings of increased jerk in autistic individuals compared to their TD counterparts (Cook et al., 2013; Ferrara et al., 2016b; Nobile et al., 2011; Torres et al., 2013; Yang et al., 2014). This study expands upon this literature on motor atypicalities in ASD to determine whether group differences exist with respect to the 2/3 PL, specifically.

Additionally, this study employed novel methodology to assess kinematics. Most research examining limb kinematics requires motion capture technology that is expensive, challenging and time-consuming to administer. In order to capture motor execution in a more cost-effective yet systematic way, there is a need for precise, digitally-based and sensitive measures which yield richer and more objective data. Digital measurement tools have also been called for by the autism community more broadly to counteract challenges associated with assessment such as the lack of measurement precision and the reliance on clinical observation (Dawson & Sapiro, 2019). Our innovative approach captured movement kinematics through an iPad activity that was accessible and appealing to children. Smart tablet technology has been used successfully to identify kinematic motor differences in ASD, with high levels of engagement amongst a large group of 3- to 6-year-old children using both machine learning and kinematic analyses (Anzulewicz et al., 2016; Lu et al., 2022; Chua et al., 2021). Novel technology holds promise as an objective, accessible, and scalable method of assessing adherence to the 2/3 PL and kinematic features.

In addition to examining adherence to the 2/3 PL specifically, we also collected more traditional measures of acceleration and jerk as well as a global measure of motor functioning. Having multiple assessments of motor performance allowed us to investigate whether individuals who showed greater divergence from the 2/3 PL in their execution of movement also showed more divergent kinematics and general motor impairment.

Given the literature on motor atypicalities in ASD and the findings of increased jerk in execution of arm movements, we predicted that autistic individuals would show greater divergence from the 2/3 PL compared to their TD counterparts, reflecting poorer motor control in accordance with this law. We also predicted that the autistic group would demonstrate greater levels of jerk, compared to the TD group. Further, we expected that metrics representing greater motor control (greater adherence to 2/3 PL, decreased jerk) would increase with age. Lastly, we hypothesized that more aberrant performance (greater deviation from 2/3 PL, greater jerk) on the iPad activity would be associated with lower fine motor skills.

## Methods

### Participants

Participants with ASD were recruited through the UC Davis MIND Institute research registry database. Participants had an existing autism diagnosis, which was obtained via clinical assessment (confirmed through clinical, medical and/or school records). TD participants were recruited through a local birth registry database (letters sent to families in areas around Davis, California, who had then agreed to participate in research). Participants were excluded from both groups if they had an acute medical condition, history of encephalopathy, seizures or traumatic brain injury, or were born more than 2 weeks prior to their due date. Additionally, autistic participants were excluded if they had another developmental or genetic condition related to ASD (e.g., fragile X syndrome, tuberous sclerosis, cerebral palsy, etc.). Participants were between ages 4 and 8, inclusive.

Participants visited the Neurocognitive Development Lab at the UC Davis Center for Mind and Brain for a 1- to 1.5-hour visit. A research team member collected informed consent from each child’s legal guardian. Verbal assent from the child was obtained, when possible. The iPad task was completed first, followed by the cognitive assessment.

### iPad Task

**Fig. 1:**
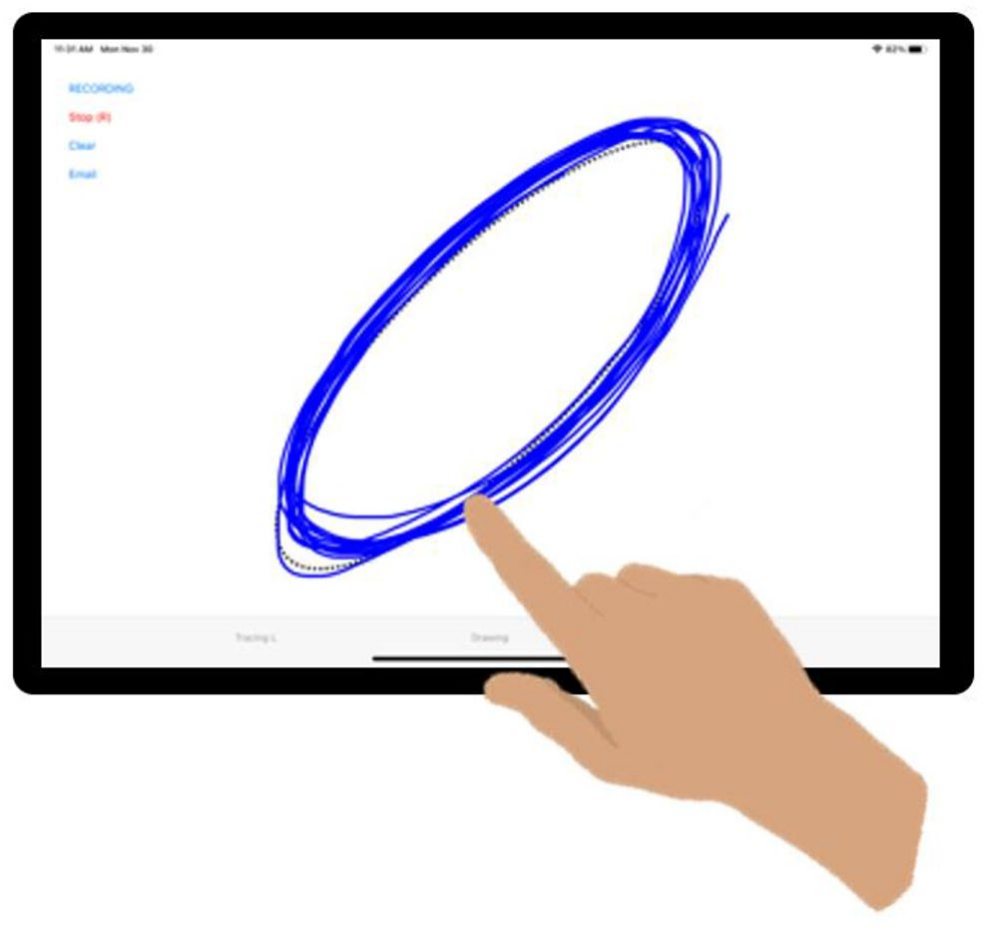
Graphic depicting the tracing activity that participants performed on the iPad tablet

To measure adherence with the 2/3 PL, we assessed the kinematics of finger movements on an iPad tablet. Participants were instructed to first draw and then trace ellipses in a bespoke application. Ellipses were chosen to build on existing literature examining the 2/3 PL developmentally. The template ellipse displayed on the screen during tracing trials had an eccentricity of 0.94, with a perimeter of 33.41 cm (although adherence to the 2/3 PL in motion production appears unaffected by eccentricity and size), and was rotated from horizontal by 45° for ease of hand movement (counter-clockwise for right-handed subjects; clockwise for left-handed subjects). See Fig. 1 for task paradigm. An example ellipse was presented to participants on a piece of paper next to the iPad while they were conducting the drawing task. Participants were free to choose the rhythm of movement but were instructed to try to maintain a constant rhythm throughout all cycles for a given trial. We included both drawing and tracing activities to examine kinematic differences resulting from varying levels of task constraints. Each participant was asked to complete 8 trials of each task, with each trial lasting about 30 s. The activity took approximately 15 min to administer.

### Behavioral Measures

We also collected a neuropsychological measure of cognitive functioning and parent-report measures of social impairment and motor ability. Overall cognitive ability was assessed using either the Wechsler Abbreviated Scale of Intelligence (WASI; Wechsler, 2011) for the first 10 participants or the Stanford-Binet Intelligence Scales, Fifth Edition (Roid & Pomplun, 2012) for all others. Both verbal and nonverbal IQ scores were obtained as well as a full-scale score. The Vineland Adaptive Behavior Scales, Second Edition (Sparrow et al., 2005) was used to assess both gross and fine motor ability. We also collected demographic information including age, race/ethnicity, gender, handedness, as well as frequency of iPad or tablet use (daily, a few times/week, few times/month, very rarely or never).

### Data Processing and Analysis

The application recorded participants’ swiping movements on the screen, collected at a sampling rate of approximately 120 Hz, in the form of x and y coordinates of finger location over time. Because the data was sampled at variable rates (due to the collection instrument and iPad ProMotion technology), x, y and time vectors were first resampled to 120 Hz. Then, x and y position vectors were filtered using a fourth-order, zero-phase shift, low-pass Butterworth filter with a cut-off frequency of 10 Hz (Bartlett, 2007). We defined continuous segments of movement as those for which a participant’s finger made constant contact with the screen; any invalid movement sections (e.g., those without end points) were excluded from analysis. We then excluded data from the first revolution of each continuous segment when participants were accelerating to reach a stable pace, as well as the final portion of movement following the last full revolution (as sampling rate often dropped off, a feature of the ProMotion technology). Resulting portions of the segments needed to include at least two full elliptical revolutions (sufficient data for linear regression) to be included in the analyses.

**Fig. 2:**
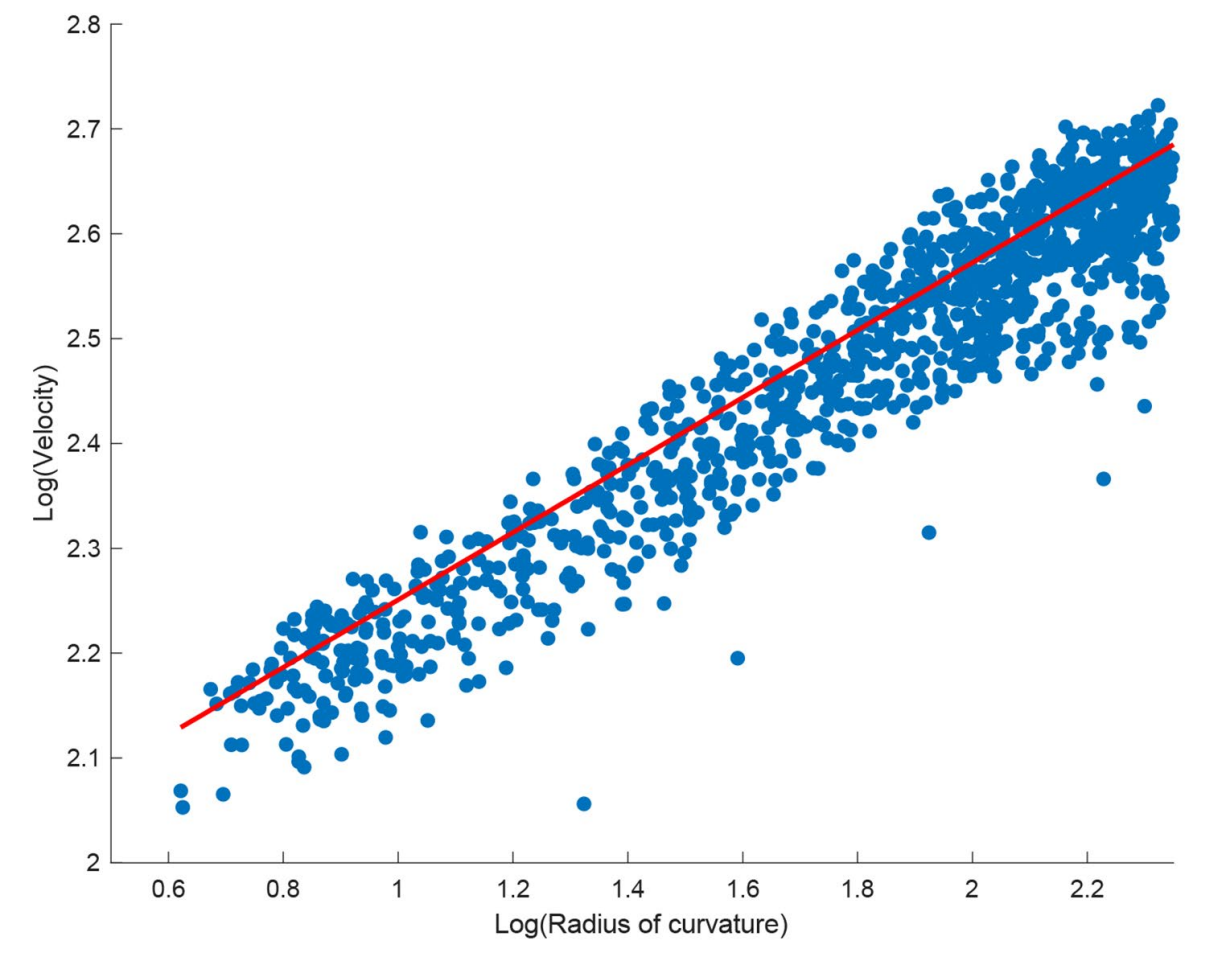
Plot of the logarithms of radius of curvature and tangential velocity for a tracing trial of a TD participant. The red line represents the slope of the relationship between the variables (shown here at β = 0.32)

To determine adherence to the 2/3 PL for each continuous segment, path curvature (measured as radius of the curvature, calculated based on three consecutive data points) and tangential velocity were computed for each data point. The common log values of these variables were linearly regressed to determine the exponent (β; beta) representing the power relation between them, according to the equation: Tangential velocity = K * Radius of curvature^β^ (see Fig. 2). Given that the 2/3 PL is demonstrated only for curvilinear trajectories, data from curvature values representing nearly straight segments (high radius of the curvature values) of movement were excluded from further analyses, a procedure that has been employed previously (Wann et al., 1988). This upper curvature threshold was determined by calculating the maximum radius of the curvature value on the template ellipse (the flattest portion): a log value of 2.35. At this point, curvature is minimal; beyond this level of flatness, we do not expect the velocity to be modulated according to the 2/3 PL. The beta exponent was calculated for data points below this curvature value cutoff.

For any continuous segment of movement, the velocity gain factor, K, is held constant and as such, irrelevant to our calculations. The exponent beta quantifies the segment’s adherence to the 2/3 PL: exponents with a value of 0.33 represent perfect adherence with the law; exponents less than 0.33 suggest that participants performed movement at more constant velocity regardless of the degree of curvature (the change in velocity across levels of curvature is less than ideal); exponents greater than 0.33 characterize movements that modulate velocity at points of curvature to a greater-than-ideal degree (the change in velocity across levels of curvature is more pronounced). Additionally, acceleration and jerk for each continuous segment (including the whole range of curvature values, even those above the 2.35 curvature cutoff) were calculated as the absolute value of the derivatives of velocity and acceleration, respectively. For each participant, the average and standard deviation of the beta exponent, acceleration and jerk across all segments was calculated separately for drawing and tracing trials.

As a further investigation to explore the difference in kinematic metrics across varying levels of curvature, we split the curvature at a level of 1.5 in logarithmic scale (based on calculations of curvature across the template ellipse; this value corresponds to a radius of curvature of 3.2 cm). Next, we calculated beta, velocity, acceleration and jerk values for data points below (more curved portions) and above (straighter portions) this cutoff point, and performed a 2 × 2 ANOVA with group (ASD vs. TD) and level of curvature (more curved vs. straighter) to explore the variability in these metrics across the varying levels of curvature.

To compare group performance on the iPad task, we used a series of t-tests or Mann-Whitney tests with each of our metrics of interest, separately for drawing and tracing trials, reporting mean value and Cohen’s d as a measure of effect size. Further, the relationship between these task-specific kinematic metrics and fine motor functioning assessed by the Vineland was examined using linear regression, to determine whether adherence to the 2/3 PL in drawing movements relates to motor functioning, more broadly. We also performed linear regression of iPad variables on age to assess the age-related trends in task-specific motor metrics.

## Results

### Sample Characteristics

The final sample included 33 TD and 24 autistic participants, ranging in age from 55 to 107 months. Groups did not significantly differ on age (*p* = .29) or performance IQ (*p* = .06). The TD group had significantly greater verbal IQ, full scale IQ and Vineland motor scores compared to the ASD group. The TD group included 16 male and 17 female participants; the ASD group included 18 male and 6 female participants. In the full sample, 38 (66%) of the participants were Caucasian, one was Asian, one was American Indian and 17 (30%) identified with multiple races. Across these racial groups, 12 participants (21%) of identified with a Hispanic/ Latinx ethnicity. See Table 1 for participant information. Within the ASD group, three participants completed only partial data collection: two completed only drawing trials, and one completed only tracing trials due to difficulties with task compliance. Based on data collection notes and visual inspection, some trials were excluded due to participants producing non-elliptical shapes.

**Table 1:** Demographics for both groups in the entire sample, including age in months, full-scale IQ (FSIQ), performance and verbal IQ, and Vineland motor subscale

	TD group	ASD group	Significance
Age (months)	80.12 (15.12)	84.46 (15.49)	*p* = .297
FSIQ	117.03 (13.17)	101.04 (17.76)	*p* < .001
Performance IQ	110.52 (12.28)	101.67 (19.21)	*p* = .06
Verbal IQ	118.33 (14.16)	98.70 (16.87)	*p* < .0001
Vineland (motor score)	104.45 (10.44)	80.26 (15.01)	*p* < .00001

### Kinematic Metrics

**Fig. 3:**
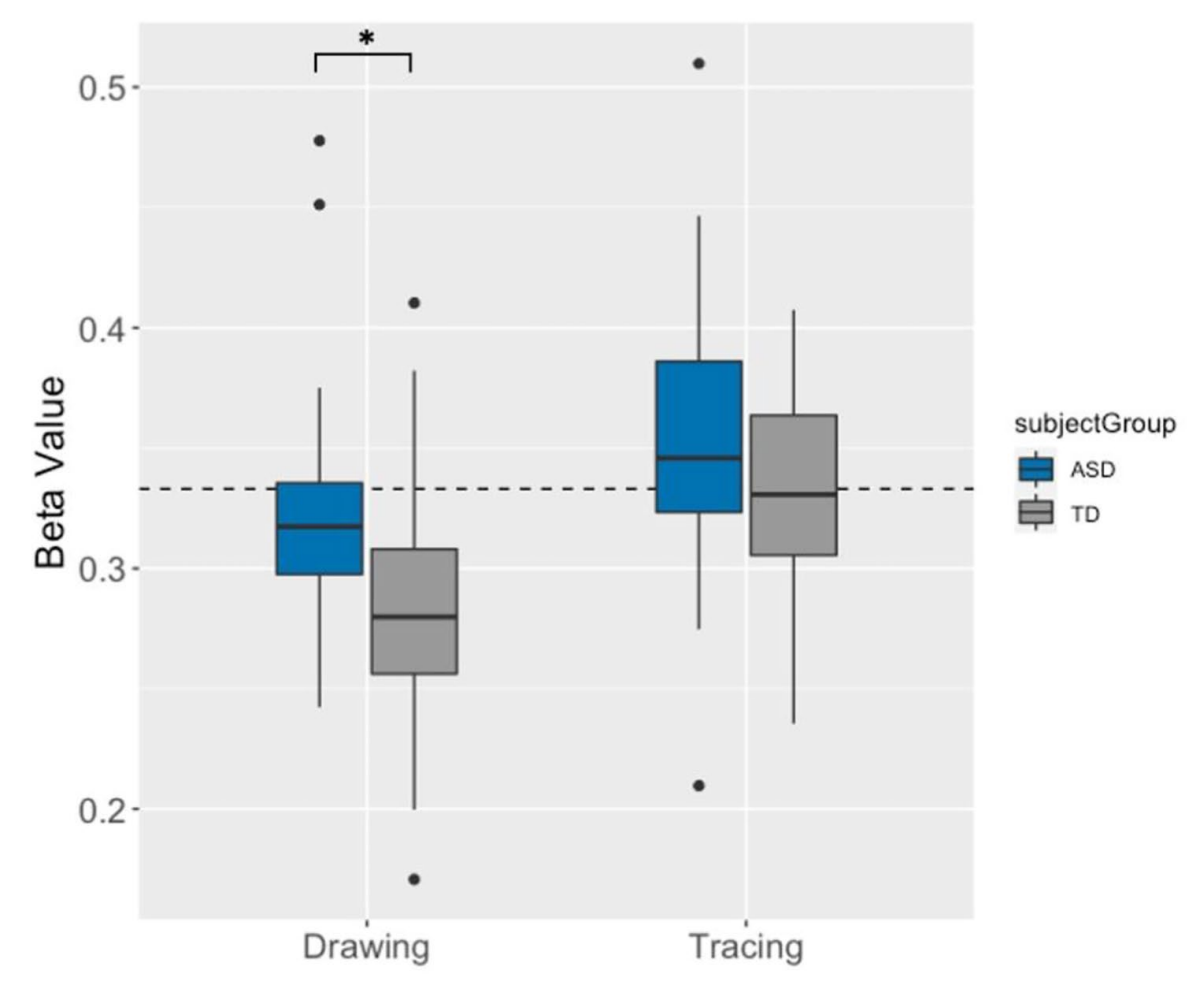
Plot of the average beta value for each trial type (drawing and tracing), by group (ASD = blue, TD = grey). The dashed line represents perfect adherence to the 2/3 PL (beta = 0.33). Significant group difference is indicated by the asterisk

On tracing trials, there was a trend toward significance (*p* = .09), with the ASD group demonstrating greater beta values, further from the 2/3 PL “ideal” value (*M* = 0.36; *M* stands for mean) compared to the TD group, which was in line with the 2/3 PL (*M* = 0.33; Cohen’s *d =* 0.46). On drawing trials, the ASD group had significantly greater beta values (*M* = 0.33), which were closer to the 2/3 PL “ideal” value than the TD group, which were lower than the “ideal value” (*M* = 0.29; *p <* .01, *d* = 0.67). See Fig. 3 for group averages of beta values for each trial type. For tracing trials, autistic participants demonstrated greater intra-individual variability across segments (*M* = 0.099), compared to TD participants (*M* = 0.063; *p* = .01). Variability on drawing trials did not significantly differ by group (TD *M* = 0.077, ASD *M* = 0.093, *p* = .4). Group variances were compared using an F-test: on tracing trials, inter-individual variance was greater in the ASD group (*M* = 0.003) compared to the TD group (*M* = 0.002; *F*_21, 32_ = 2.18, *p* = .045). On drawing trials, group variances did not differ (*F*_22, 32_ = 1.00, *p* = .96).

**Fig. 4:**
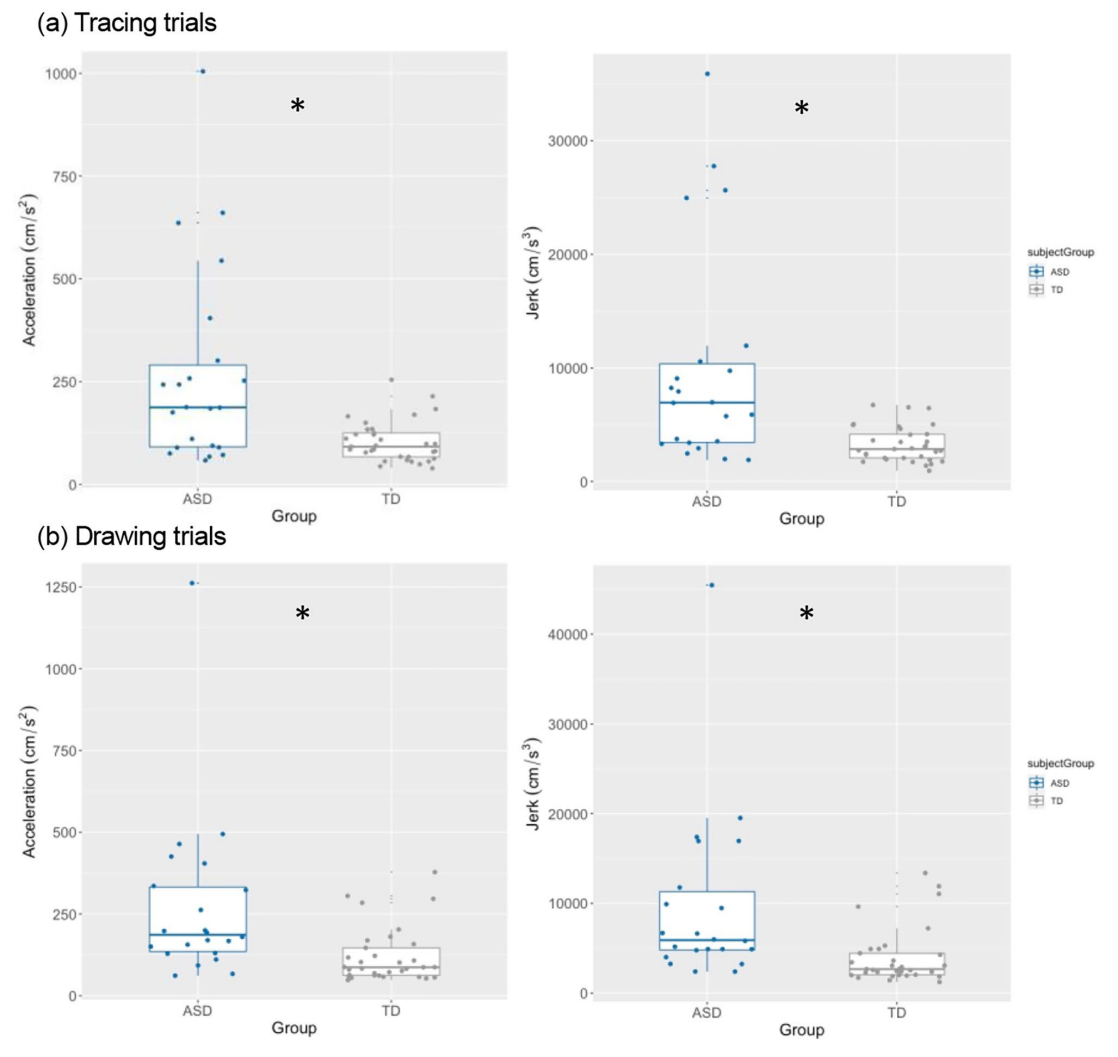
Average acceleration and jerk for both (a) tracing and (b) drawing trials across both groups (ASD = blue; TD = grey). Dots depict individual participant average values. An asterisk represents a significant group difference

Metrics of absolute acceleration and jerk were also examined. Compared to TD participants, autistic participants showed significantly greater acceleration during drawing (ASD *M* = 271.71, TD *M* = 120.43, *p* = .0001, *d* = 0.78) and tracing trials (ASD *M* = 269.81, TD *M* = 103.55, *p* = .0007, *d =* 0.94). They also showed greater levels of jerk on both drawing (ASD *M* = 9663.98, TD *M* = 3996.15, *p* < .0001, *d =* 0.78) and tracing trials (ASD *M* = 9578.42, TD *M* = 1556.27, *p* < .0001, *d* = 0.98). Additionally, the variance in both metrics was significantly greater in the ASD group compared to the TD group on both tracing (both *p* < .00001) and drawing trials (both *p* < .00001). See Fig. 4. Despite group differences in IQ, there were no associations between IQ and any kinematic metric, and thus no further analyses were conducted to control for IQ as a covariate.

Given that group differences emerged across all kinematic metrics, we wanted to explore whether these differences would remain when controlling for the other kinematic variables, particularly beta value and jerk because they are similar constructs, both capturing smoothness of movement. We performed ANCOVAs to examine group differences with the other metric included as a covariate, for both drawing and tracing trials. All group differences remained significant (*p* < .01; only beta value on tracing trials did not show a significant result, but the group difference was not significant initially as noted above).

### Curvature Split Analysis

To further explore the role of group differences and examine whether more curved (low radius of curvature) or straighter (high radius of curvature) portions of movement were driving group differences, we examined whether kinematic metrics (beta value, velocity, acceleration, jerk) varied as a function of curvature differently across groups using a 2 × 2 ANOVA with group (ASD vs. TD) and level of curvature (straighter/high radius vs. curved/low radius).

**Fig. 5:**
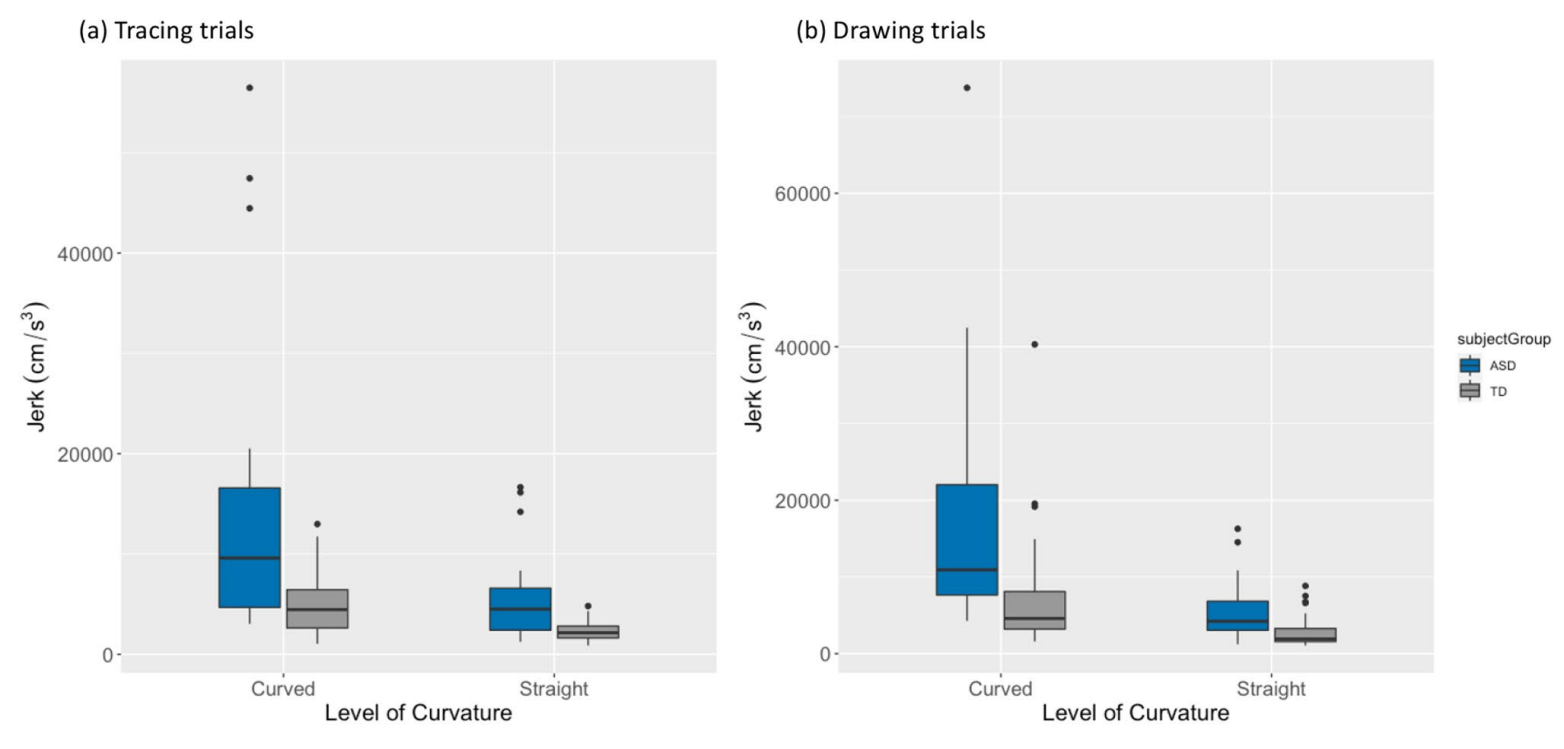
Average jerk by curvature level and group (ASD = blue, TD = grey), on (a) tracing and (b) drawing trials. Critically, on both tracing and drawing trials, there was an interaction effect such that differences in jerk across curvature levels were more different in the ASD compared to the TD group

On tracing trials, beta values showed a significant effect of curvature, but no main effect of group or interaction. However, average beta values for groups differed by a larger degree on more curved portions of movement (ASD *M* = 0.39, TD *M* = 0.36) compared to the straighter portions (ASD *M* = 0.26, TD *M* = 0.25), despite not reaching significance. For velocity, there was a main effect of group (*p* = .002), a main effect of curvature (*p* < .000001), and an interaction between group and curvature (*p* = .002). Pairwise comparisons revealed that velocity was faster on straighter compared to the more curved sections (as is dictated by the 2/3 PL), and in the ASD compared to the TD group overall. Notably, the interaction effect was driven by a *greater* difference in velocity between straighter and more curved portions of movement in the ASD group compared to the TD group. The same 2 × 2 ANOVA for acceleration resulted in only a main effect of group (*p* = .007), such that the ASD group demonstrated greater acceleration than the TD group across both levels of curvature, but neither the effect of curvature (*p* = .08) nor the interaction between group and curvature (*p* = .21) was significant. Jerk values also showed a main effect of group (*p* = .0004), main effect of curvature (*p* < .000001), and an interaction between group and curvature (*p* = .001). Similar to velocity, the significant interaction was a result of the ASD group showing a *greater* difference in jerk between straighter and more curved portions of movement compared to the TD group. See Fig. 5.

On drawing trials, there was a significant effect of curvature (*p* < .00001) and marginally significant effect of group (*p* = .059) on beta value, such that average beta values on straighter portions of movement were significantly greater in the ASD (*M* = 0.23) compared to TD group (*M* = 0.19; *p* = .028), while more curved portions did not differ by group (ASD *M* = 0.35, TD *M* = 0.34, *p =* .81). For velocity, there was main effect of both group (*p* = .002) and curvature (*p* < .00001) as well as an interaction between them (*p* = .002). Pairwise comparisons show that the ASD group had greater differences in velocity between more curved and straighter portions compared to the TD group. Acceleration showed a main effect of group (*p* = .0007), a main effect of curvature (*p* < .00001), but no interaction. Lastly, jerk values showed a main effect of group (*p* < .00001; ASD > TD), a main effect of curvature (*p* < .00001; curved > straight) and an interaction effect (*p* = .004), in which the difference in jerk between more curved and straighter sections was more pronounced in the ASD group.

### Age-Related Trends in Kinematic Features

**Fig. 6:**
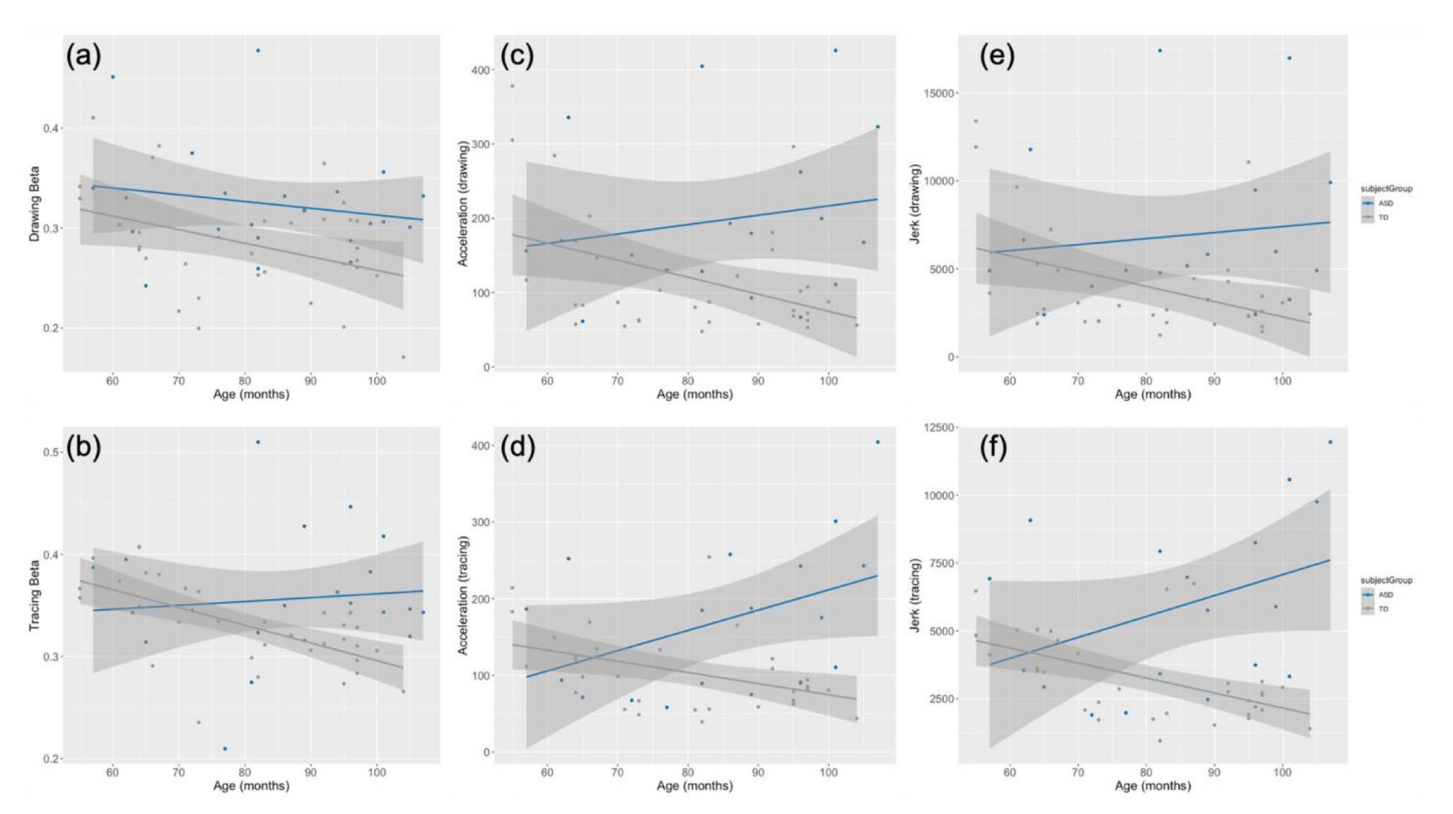
Developmental trends by group (ASD = blue, TD = grey) in beta value (**a**, **b**) , acceleration (**c**, **d**) and jerk (**e**, **f**) for both trial types

Linear regressions of motor kinematic variables with age were performed (Fig. 6). For tracing trials, the TD group showed a negative association between age and beta value (*r* = − .002, *p* < .0001), acceleration (*r* = -1.45, *p* = .01) and jerk (*r* = -55.21, *p* = .001). Similarly, on drawing trials, there was a significant negative relationship between age and beta value (*r* = − .001, *p* = .028), acceleration (*r* = -2.29, *p* = .02) and jerk (*r* = -86.50, *p* = .016) in the TD group. There were no significant relationships between age and any kinematic features in the ASD group.

### Relationship with Motor Functioning

Finally, we examined the relationship between metrics on the iPad task (beta value, individual variability in beta value and jerk) and parent-reported motor skills as measured by the Vineland Adaptive Behavior Scales. We focused on the fine motor v-scale score, as the iPad task more heavily utilized fine motor skills. The only significant association that emerged was a negative relationship between variability in beta value and fine motor score (*r* = − .01, *p* = .01), such that less intra-individual variability across drawing trials was related to greater motor functioning. No other associations reached significance. In addition, no kinematic metrics on the task were related to frequency of iPad/tablet use.

## Discussion

### Adherence to the 2/3 PL

Using novel iPad methodology, this study examined whether autistic and TD participants differ in how well their drawing movements adhered to the 2/3 PL. This law of motion models a mathematically prescribed coupling between the curvature and velocity of a movement and governs drawing movements from an early age. This task was successful in producing movement with this characteristic curvature-velocity coupling, with average participant beta values ranging from 0.2 to 0.5 (0.33 represents perfect adherence to the 2/3 PL). On both drawing and tracing trials, our task revealed group differences in beta value, with the ASD group showing higher beta values. Further, these differences remained when accounting for jerk, a related metric, suggesting that beta and jerk capture unique aspects of motor control.

Tracing trials presented a more constrained task, where participants moved along a prescribed elliptical trajectory, yielding results that are more comparable across both trials and participants. On tracing trials, the TD group’s beta value of 0.33 suggests close adherence to the 2/3 PL, while the increased beta value in the ASD group (*M* = 0.36) points to an atypical velocity–curvature coupling. Specifically, the elevated beta value in the ASD group suggests that the finger’s change in velocity across the range of curvature is steeper than “ideal” (i.e., perfect adherence to 2/3 PL). This would indicate that velocity slows by more than is “ideal” on more curved sections, while velocity increases by more than is “ideal” in straighter sections. The beta values observed across groups in our study are higher than what has been shown in a previous study using a similar protocol with template ellipses, in the range of 0.27 to 0.30 for children ages 5 to 8 (Viviani & Schneider, 1991). It should be noted that we used higher data sampling rates (120 Hz in our task vs. 88 Hz in the previous study) and a filtering protocol (no filtering procedure was reported in Viviani & Schneider, 1991).

Drawing trials, on the other hand, presented a much less constrained, free-form assessment of motor performance. During these trials, participants often produced drawings that differed in size and shape and were highly variable across trials, since there was no template to follow. These trials also revealed greater beta values in the ASD (*M* = 0.33) compared to the TD group (*M* = 0.29), however the ASD group actually demonstrated closer adherence to the 2/3 PL. While a beta value of 0.33 is considered to represent the perfect adherence to the 2/3 PL, it may not be appropriate to evaluate against this standard metric on drawing trials. While the same elliptical shape was presented in both trial types, on drawing trials participants copied it without a template on screen and often had difficulty accurately extending the full length of the ellipse. Thus, the shapes produced on drawing trials tended to be more circular in shape. As such, data collected on these trials did not have as large a range of curvature values, and was especially limited in the most curved portions. As was revealed by the curvature split analysis, beta values are higher in the curved compared to straight sections of movement, thus with fewer data points in the most curved range of values, a lower beta value would be expected. Indeed, the beta values produced by the drawing task were lower than those in the tracing task across both groups (Fig. 3). It is more likely that the ASD group’s average beta value of 0.33 from the drawing trials is a reflection of this limitation, rather than a result of those with ASD exhibiting closer adherence to the law. What is most critical is that group differences in the same direction (ASD > TD) were observed for both trial types. This is the first study to investigate adherence to the 2/3 PL in autistic individuals and has demonstrated that those with ASD appear to show a steeper velocity-curvature coupling than their TD counterparts across both tracing (highly constrained) and drawing (less constrained) trials.

In order to better interpret these group differences in the beta value, we performed a split of the curvature variable at a pre-determined value of 1.5 (which corresponds to a relative cut-off between straight and curved sections of movement) for kinematic variables of velocity, acceleration and jerk. Both drawing and tracing trials showed similar trends and will be discussed together. For velocity, the main effect of curvature level (curved vs. straight) was expected based on the relationship modeled by the 2/3 PL equation, such that velocity was greater on the straighter compared to more curved portions. It also appears that the ASD participants moved more quickly across the entirety of the movement compared to their TD counterparts across both trial types. Given that participants were instructed to choose whatever speed felt most comfortable to them, autistic participants may have simply preferred a faster speed. This finding supports previous literature showing that autistic individuals write and draw with higher velocity (Grace et al., 2017; Lu et al., 2022), however this finding may be task dependent. Most important for parsing the group differences in beta value was the interaction between curvature level and group in velocity, significant in both drawing and tracing trials. This interaction effect was driven by a greater difference in velocity between straighter and more curved portions of movement in the ASD group compared to the TD group. In other words, the ASD group modulated their velocity to a greater degree based on the curvature of the movement than did the TD group. This is consistent with the interpretation of the elevated beta value across the entire segment (i.e., a higher/steeper beta value means greater modulation of velocity along the curvature range).

While the actual group differences in beta values are small, these exploratory analyses provide some information about what is driving these differences. The 2/3 PL prescribes a close relationship between curvature and velocity in curvilinear movements, and indeed, we see this coupling between variables in both groups. However, it appears that autistic participants’ movements resulted in higher beta values because their modulation of velocity between straighter and more curved portions of movement is more pronounced than it is in TD participants. The presence of this same characteristic during both drawing and tracing suggests that the way in which velocity is modulated may be inherent to autism, irrespective of the task constraints. This finding also points to a potential factor underlying the atypical motor control across a range of activities in ASD.

### Acceleration and Jerk

In addition to our primary aim assessing adherence to the 2/3 PL in movement, we also examined kinematic metrics of acceleration and jerk. These are more commonly studied and generalizable characteristics of movement which allow us compare results from this study to other literature on motor control in ASD that employed these same metrics. Our task revealed group differences in jerk and acceleration, with ASD participants showing greater levels of acceleration and jerk on both drawing and tracing trials. These group differences in jerk remained significant when controlling for beta value, suggesting an independence of these constructs. Jerk is the third time derivative of (finger) position, the rate of change of acceleration. Lower jerk is associated with better control and greater smoothness of movement (Todorov & Jordan, 1998). It can be thought of analogously in terms of driving, wherein a driver with more experience is able to accelerate and decelerate the vehicle smoothly, whereas a less experienced driver may produce a *jerkier* ride. It has also been investigated as a key factor influencing whether movements are perceived as natural. A study by Aransih and Edison (2019) found that jerk was the most significant factor in the perception of movement naturalness. Greater levels of jerk resulted in movements that appeared less natural, in contrast to smooth and controlled movements.

The finding of increased jerk seems to be observed consistently in the literature on motor production in autism, across both gross and fine motor movements (Cook et al., 2013; Ferrara et al., 2016b; Nobile et al., 2011; Torres et al., 2013; Yang et al., 2014). These studies employed different paradigms, methods of movement capture and types of movements, so replicating the finding of increased jerk in our study expands upon this growing body of research. Furthermore, our results align with other studies that have examined other components of movement which are not explicitly labelled as jerk, but likely related. Research has shown that autistic children make more corrective adjustments (also known as movement units) during the execution of a goal-directed task (Anzulewicz et al., 2016; Forti et al., 2011; Chua et al., 2021; Whyatt & Craig, 2013). If greater jerk (or acceleration) is conceptualized as more changes in acceleration (or velocity), greater corrective movements may similarly represent this pattern of increased jerk. These findings overall point to a tendency for less efficiency in motor planning and execution in autism.

Inter-individual variability in jerk and acceleration was also increased in the ASD group, suggesting that autistic individuals varied more in the amount of acceleration and jerk with which they performed these movements. This tracks with the greater heterogeneity that is commonly observed in autistic samples. Visual inspection of tracing data (Fig. 4[a]) reveals four outliers in the ASD group with elevated levels of *both* acceleration (above 500 cm/Sect. ^2^) and jerk (above 25,000 cm/Sect. ^3^). These participants may represent a subset of autistic individuals with highly atypical movement kinematics. Although these outliers have contributed to the elevated average across the autistic group, they do not solely account for the increased levels of acceleration and jerk in the autistic group, as the group difference remains significant even when they are removed from analyses.

In addition to examining these metrics across the entirety of the ellipses, the curvature split procedure was used to explore how the kinematics of movement change across the duration of the elliptical drawing and revealed differential results for acceleration and jerk metrics. For both acceleration and jerk, there were main effects of group and curvature level: both metrics were greater in more curved compared to straighter sections, and for autistic compared to TD participants. However, only jerk, but not acceleration, showed an interaction between curvature level and group, such that jerk increased by a greater degree on more curved versus straighter sections in the ASD group, as compared to the TD group. This finding indicates that jerk, more so than acceleration, may be a particularly important indicator of motor dysfunction in ASD. This finding could have implications for writing and drawing where the majority of movements performed are curved. If autistic individuals show increased jerk when performing curved movements, whether on a tablet, paper or in space, this would likely correspond to difficulties with motor control across a range of activities.

Findings from this iPad assessment related to acceleration and jerk, as well as adherence to the 2/3 PL, reveal clear differences between ASD and TD groups, suggesting that this novel methodology can provide useful insight by quantifiably characterizing the atypicalities of drawing movements in autistic individuals. It also helps to solve a methodological issue in the field, by objectively capturing movement kinematics in a way that circumvents the need for expensive and difficult-to-administer motion capture technology, while remaining fun and engaging for children.

### Relationship between Kinematic Measures and Age

We also explored whether age showed any association to beta value (adherence to 2/3 PL) or other kinematic variables to determine whether these metrics capturing motor control would show a developmental progression. Given research that suggests early adherence to the 2/3 PL which may be further tuned by experience across development, as well as an understanding that motor control (which the 2/3 PL indexes) improves across development, we expected to observe an increase in beta value with age. On the contrary, we observed a negative association between beta value and age, only in the TD group, such that older children showed lower beta values. This finding contradicts the only previous study that examined beta value as a function of age using a similar ellipse tracing task, which revealed a positive association (Viviani & Schneider, 1991). The authors found that across their sample of children ages 5 to 12, the beta value increased toward the “ideal” or adult level of 0.33, except the 5-year-olds did not fit the age-related trend. However, this previous study did not look at age continuously, but in distinct age brackets with only 6 individuals in each, and in doing so, may have missed a more fine-grained relationship between age and beta value. If we inspect our data visually, it does appear that the beta values derived from tracing trials hover closer to 0.33 for older TD children (Fig. 6[b]), while they appear to be elevated in younger children (mostly in the range of 0.34 to 0.40; leading to the negative association). The fact that most of the younger TD participants and nearly all autistic participants show the same trend of beta value above 0.33 suggests that an elevated beta value could represent a delay in motor control, present in younger and autistic participants. Furthermore, age-related trends in the beta value were only observed in TD but not ASD, indicating developmental stasis in ASD across this age range. Yet, it should be noted that this analysis was conducted on a cross-sectional dataset. Given that the range included in our sample was not designed to assess age, further studies with larger sample sizes would allow for better exploration of the developmental trajectories of this power law.

For more traditional metrics of jerk and acceleration during the drawing/tracing task, we expected an age-related decrease in these kinematic features. Indeed, both metrics significantly decreased with age across both drawing and tracing trials, in the TD group only (Fig. 6). In general, smooth and controlled movement is associated with smaller higher-order derivatives (i.e., acceleration, jerk; Todorov & Jordan, 1998), so this finding suggests there was better motor control on this task with increasing age. Yet, this age-related trend was not present in the ASD group, which could indicate that the typical developmental progression of motor control is disrupted in autism.

### Association with Motor Functioning

As part of this study, we hypothesized that variables of interest collected during the iPad activity would be related to motor functioning. For the most part, we did not find evidence across the various iPad metrics of such an association. However, one significant relationship emerged: less intra-individual variability in beta value was associated with better motor functioning. This suggests that consistency across trials may be an important predictor of motor outcomes. Contrary to our expectations, it does not appear that the task specific metrics of beta value and jerk are linked to global fine motor skills. There are several possible reasons for this. First, the v-scale score of the fine motor subdomain has a narrow range (7 to 21 across the whole sample), and within each group, ranges are even narrower. Additionally, fine motor skills were assessed by parent report through questions related to the use of scissors, coloring, and drawing letters and shapes, skills which are more commonly demonstrated in school and could potentially be better captured by teacher report. Alternatively, using a direct motor assessment such as the Movement Assessment Battery for Children or the Bruininks-Oseretsky Test of Motor Proficiency may have been more successful at detecting relationships between task-specific motor performance and global motor functioning. Lastly, our sample size may have been too small to detect an effect.

### Limitations and Future Directions

There are a number of limitations to this study as well as areas for further exploration. All analyses would benefit from larger sample sizes, especially in the ASD group. Autism is known to be heterogeneous and this was reflected in the increased variability across nearly every metric assessed. Recent research approaches have taken to parsing this heterogeneity by identifying subgroups with differential behavioral or biological signatures. Future investigations may benefit from such an approach but would require much larger sample sizes. A related approach would be to examine whether any of the task metrics track with autism symptomology. However, due to testing limits during the COVID-19 pandemic, we relied on existing diagnostic reports and did not have ADOS scores available for all participants to use in this way. It is also important to note that while this study observed age-related changes in our TD cohort, this is a cross-sectional study and is not likely to provide an accurate depiction of how adherence to this law progresses developmentally. In addition to larger sample sizes, longitudinal work would be needed to fully assess these trajectories of motor development.

Additionally, groups differed in IQ: the TD group had higher full-scale and verbal IQ and a trend toward higher performance IQ. Despite these group differences, no associations between IQ and any kinematic metric emerged in the analyses. These null findings may suggest that motor delays observed in ASD are independent of cognitive delays and such a methodological approach may prove useful in disentangling the motor component of development from cognition. Despite the lack of statistical evidence, it is still possible that cognitive level influenced performance on the task. We attempted to include participants with a wide range of cognitive abilities and did not exclude based on IQ, however, the tasks did require a certain level of verbal comprehension and attention, making it challenging for those with low cognitive functioning or intellectual disability to participate. As such, it is difficult to extrapolate the findings to autistic individuals who are entirely non-verbal or who have high support needs. It may be useful for future studies to include another comparison group, with more closely matched IQ and/or other developmental delay to determine whether observed differences in perceptual and motor performance are specific to autism or related to more general cognitive functioning.

The iPad task also presented some limitations. First, the elliptical shape drawn by participants is not common to everyday movements and therefore lacked full ecological validity. For the purposes of investigating adherence to the 2/3 PL, it was important to use an ellipse both to build upon prior literature and to standardize across participants. However, other studies assessing motor movements using more ecologically valid approaches show the same velocity-curvature relationship (Adi-Japha et al., 1998; Fetters & Todd, 1987; von Hofsten & Rönnqvist, 1993). Thus, we would hypothesize that the findings observed in our task, with respect to the power law and jerk, are likely to extend to other curvilinear movements.

## Conclusion

This novel iPad task provided some unique insight into the kinematic differences of movements generated by autistic individuals. Technology like this is being used more frequently to systematically assess various features of motor performance, as it provides a more precise, objective, and quantifiable measure of motor functioning, beyond neuropsychological assessments or parent report measures. A similar approach using different drawing games on an iPad was successfully able to identify distinct patterns of movement kinematics, an “autism motor signature,” using machine learning algorithms and kinematic analyses (Anzulewicz et al., 2016; Lu et al., 2022; Chua et al., 2021). While the diagnostic utility of a tool like this remains under consideration, it nevertheless presents opportunities to better understand the motor atypicalities present in autism, especially early in development. Further development and clinical testing may yield an accessible, scalable early identification tool for clinical diagnosticians or screening programs (Millar et al., 2019).

Improved knowledge of motor differences in autism and computational characterization of the so-called “autism motor signature” can offer potential for more tailored motor intervention. As previously discussed, a large majority of autistic children present with motor impairment, but many go undiagnosed and untreated (Bhat, 2020). A sensitive computational assessment like this may be able to detect specific motor profiles or identify those with the most divergent kinematic profiles (e.g., the autistic outliers with jerk > 25,000 cm/s^3^ in Fig. 4), and implement more targeted, individualized intervention in order to improve motor skills and bolster motor learning (Holloway et al., 2022), with downstream gains in social and cognitive skills. Further exploration is required to determine whether these features are consistent with motor delays more generally, and what the underlying mechanisms leading to this disruption may be.

In this paper, we have identified motor control differences in a simple curvilinear motor control task. These differences reflect autistic differences in a fundamental aspect of skilled motor performance important for tasks as common as writing and drawing, on which all school learning and assessment is based, and we can expect that these performance differences on a tablet screen translate to functional and expressive movements in 3D space such as walking, reaching-to-grasp, and gesture in communication. Altogether, these data show how basic motor kinematics are disrupted, leading to consequential experience-dependent growth in communication and learning. We further show age-related changes evident in TD children are not evident in ASD children, suggesting a fundamental neuromotor age-independent disruption, or developmental stasis. The lack of age-related changes in ASD may be related to motor learning difficulties or developmental delay in ASD, but a growing alternative explanation suggests these aspects of the autism motor signature are age-independent and pre-date motor skill acquisition. Their source appears to rest within more basic, ontogenetically prior neuromotor mechanisms mediated by the brainstem sensorimotor integration (Dadalko & Travers, 2018; Delafield-Butt et al., 2022; Delafield-Butt & Trevarthen, 2017; Travers et al., 2015; Trevarthen & Delafield-Butt, 2013). Finally, we demonstrate the potential of a useful new methodology that may ultimately contribute to further computational characterization of the autism motor signature for early, accessible, and scalable assessment in screening or diagnostic pathways.

## Footnotes

1 A non-peer-reviewed preprint became available during the revisions of this manuscript that investigated power law differences in adults with autism, of interest here (Cook et al., 2023).

## References

[CR1] Adi-Japha, E., Levin, I., & Solomon, S. (1998). Emergence of representation in drawing: The relation between kinematic and referential aspects. *Cognitive Development*, *13*(1), 25–51. 10.1016/S0885-2014(98)90019-3.

[CR2] American Psychiatric Association. (2013). *Diagnostic and statistical Manual of Mental disorders*. American Psychiatric Association. 10.1176/appi.books.9780890425596.

[CR3] Anzulewicz, A., Sobota, K., & Delafield-Butt, J. T. (2016). Toward the Autism Motor signature: Gesture patterns during smart tablet gameplay identify children with autism. *Scientific Reports*, *6*(1), 31107. 10.1038/srep31107. 27553971 10.1038/srep31107 PMC4995518

[CR4] Aransih, M. P., & Edison, R. E. (2019). The naturalness of Biological Movement by individuals with Autism Spectrum conditions: Taking neurotypical individuals’ viewpoint. *Neurology and Psychiatry Open Access Maced J Med Sci*, *7*(16), 2574. 10.3889/oamjms.2019.392. 10.3889/oamjms.2019.392 PMC6876820 31777608

[CR5] Bartlett, R. (2007). *Introduction to sports Biomechanics: Analysing Human Movement patterns*. Routledge.

[CR6] Beversdorf, D. Q., Anderson, J. M., Manning, S. E., Anderson, S. L., Nordgren, R. E., Felopulos, G. J., & Bauman, M. L. (2001). Brief report: Macrographia in high-functioning adults with autism spectrum disorder. *Journal of Autism and Developmental Disorders*, *31*(1), 97–101. http://www.ncbi.nlm.nih.gov/pubmed/11439759. 11439759 10.1023/a:1005622031943

[CR7] Bhat, A. N. (2020). Is motor impairment in Autism Spectrum disorder distinct from Developmental Coordination Disorder? A Report from the SPARK Study. *Physical Therapy*, *100*, 1–12. 10.1093/ptj/pzz190/5801997. 32154876 10.1093/ptj/pzz190 PMC7297441

[CR8] Bhat, A. N. (2021). Motor Impairment increases in Children with Autism Spectrum Disorder as a function of Social Communication, cognitive and functional impairment, repetitive behavior severity, and Comorbid diagnoses: A SPARK study report. *Autism Research*, *14*(1), 202–219. 10.1002/AUR.2453. 33300285 10.1002/aur.2453 PMC8176850

[CR9] Bidet-Ildeil, C., Orliaguet, J. P., Sokolov, A. N., & Pavlova, M. (2006). Perception of elliptic biological motion. *Perception*, *35*(8), 1137–1147. 10.1068/P5482. 17076071 10.1068/p5482

[CR10] Bishop, S. L., Wickstrom, J., & Thurm, A. (2022). Insufficient evidence for inclusion of motor deficits in the ASD diagnostic criteria: A response to Bhat (2021). *Autism Research: Official Journal of the International Society for Autism Research*, *15*(8), 1374–1375. 10.1002/AUR.2775. 35779237 10.1002/aur.2775

[CR11] Bondioli, M., Chessa, S., Narzisi, A., Pelagatti, S., & Zoncheddu, M. (2021). Towards motor-based early detection of autism red flags: Enabling technology and exploratory study protocol. *Sensors (Basel, Switzerland)*, *21*(6), 1–15. 10.3390/s21061971. 10.3390/s21061971 PMC7998381 33799643

[CR12] Calhoun, M., Longworth, M., & Chester, V. L. (2011). Gait patterns in children with autism. *Clinical Biomechanics*, *26*(2), 200–206. 10.1016/J.CLINBIOMECH.2010.09.013. 20934239 10.1016/j.clinbiomech.2010.09.013

[CR13] Casartelli, L., Molteni, M., & Ronconi, L. (2016). So close yet so far: Motor anomalies impacting on social functioning in autism spectrum disorder. *Neuroscience & Biobehavioral Reviews*, *63*, 98–105. 10.1016/J.NEUBIOREV.2016.02.001. 26855233 10.1016/j.neubiorev.2016.02.001

[CR14] Casile, A., Dayan, E., Caggiano, V., Hendler, T., Flash, T., & Giese, M. A. (2010). Neuronal Encoding of Human Kinematic Invariants during Action Observation. *Cerebral Cortex*, *20*(7), 1647–1655. 10.1093/cercor/bhp229. 19933580 10.1093/cercor/bhp229

[CR15] Cho, A., Bin, Otte, K., Baskow, I., Ehlen, F., Maslahati, T., Mansow-Model, S., Schmitz-Hübsch, T., Behnia, B., & Roepke, S. (2022). Motor signature of autism spectrum disorder in adults without intellectual impairment. *Scientific Reports*, *12*(1). 10.1038/S41598-022-10760-5. 10.1038/s41598-022-10760-5 PMC9090847 35538115

[CR16] Chua, Y., Lu, S. C., Anzulewicz, A., Sobota, K., Tachtatzis, C., Andonovic, I., Rowe, P., Delafield-Butt, J., & Chua, Y. W., C (2021). Developmental differences in the prospective organisation of goal-directed movement between children with autism and typically developing children: A smart tablet serious game study. *Developmental Science*, e13195. 10.1111/DESC.13195. 10.1111/desc.13195 PMC9287065 34800316

[CR17] Cook, J. L., Blakemore, S. J., & Press, C. (2013). Atypical basic movement kinematics in autism spectrum conditions. *Brain*, *136*(9), 2816–2824. 10.1093/brain/awt208. 23983031 10.1093/brain/awt208 PMC4017873

[CR18] Cook, J. L., Fraser, D. S., Hickman, L. J., & Brewer, R. (2023). *Autistic kinematics diverge from the power laws that typically govern movement*. 1–35.

[CR19] Crippa, A. (2022). Motor abilities as a possible specifier of autism: A response to Bhat (2021). *Autism Research*. 10.1002/AUR.2805. 36036959 10.1002/aur.2805

[CR20] Cummins, A., Piek, J. P., & Dyck, M. J. (2007). Motor coordination, empathy, and social behaviour in school-aged children. *Developmental Medicine & Child Neurology*, *47*(7), 437–442. 10.1111/j.1469-8749.2005.tb01168.x. 10.1017/s001216220500085x 15991862

[CR21] Dadalko, O. I., & Travers, B. G. (2018). Evidence for brainstem contributions to autism spectrum disorders. *Frontiers in Integrative Neuroscience*, *12*(October), 1–17. 10.3389/fnint.2018.00047. 30337860 10.3389/fnint.2018.00047 PMC6180283

[CR22] Dawson, G., & Sapiro, G. (2019). Potential for Digital Behavioral Measurement Tools to transform the detection and diagnosis of Autism Spectrum Disorder. *JAMA Pediatrics*, *173*(4), 305–306. 10.1001/JAMAPEDIATRICS.2018.5269. 30715131 10.1001/jamapediatrics.2018.5269 PMC7112503

[CR23] Dayan, E., Casile, A., Levit-Binnun, N., Giese, M. A., Hendler, T., & Flash, T. (2007). Neural representations of kinematic laws of motion: Evidence for action-perception coupling. *Proceedings of the National Academy of Sciences of the United States of America (Vol*, *104*(51). 10.1073/pnas.0710033104. 10.1073/pnas.0710033104 PMC2154474 18079289

[CR24] De’Sperati, C., & Viviani, P. (1997). The relationship between curvature and velocity in two-dimensional smooth pursuit eye movements. *The Journal of Neuroscience: The Official Journal of the Society for Neuroscience*, *17*(10), 3932–3945. 10.1523/JNEUROSCI.17-10-03932.1997. 9133411 10.1523/JNEUROSCI.17-10-03932.1997 PMC6573701

[CR26] Delafield-Butt, J., & Trevarthen, C. (2017). On the brainstem origin of autism disruption to movements of the primary self. In E. Torres, & C. Whyatt (Eds.), *Autism: The Movement sensing perspective*. Taylor & Francis CRC Press. Issue February10.1201/9781315372518.

[CR25] Delafield-Butt, J., Dunbar, P., & Trevarthen, C. (2022). Disruption to the Core Self in Autism, and its care. *Psychoanalytic Inquiry*, *42*(1), 53–75. 10.1080/07351690.2022.2007031.

[CR27] Dewey, D., Cantell, M., & Crawford, S. G. (2007). Motor and gestural performance in children with autism spectrum disorders, developmental coordination disorder, and/or attention deficit hyperactivity disorder. *Journal of International Neuropsychological Society*, *13*, 246–256. 10.1017/S1355617707070270. 10.1017/S1355617707070270 17286882

[CR28] Ekberg, T. L., Falck-Ytter, T., Bölte, S., Gredebäck, G., Campbell, L., Cauvet, E., Kleberg, J. L., Jobs, E. N., Nyström, P., Thorup, E., & Zander, E. (2016). Reduced prospective motor control in 10-month-olds at risk for autism spectrum disorder. *Clinical Psychological Science*, *4*(1), 129–135. 10.1177/2167702615576697.

[CR29] Ferrara, M., Zuccalá, V. C., Cecchi, F., Laschi, C., Delafield-Butt, J. T., & Passetti, G. (2016a). *Motor kinematic differences in children with autism sepectrum disorder: ecological gameplay with a sensorised toy*. https://strathprints.strath.ac.uk/58580/.

[CR30] Ferrara, M., Zuccalá, V. C., Cecchi, F., Laschi, C., Delafield-Butt, J. T., & Passetti, G. (2016b). *Motor kinematic differences in children with autism sepectrum disorder: ecological gameplay with a sensorised toy*. https://strathprints.strath.ac.uk/58580/.

[CR31] Fetters, L., & Todd, J. (1987). Quantitative assessment of infant reaching movements. *Journal of Motor Behavior*, *19*(2), 147–166. 10.1080/00222895.1987.10735405. 14988056 10.1080/00222895.1987.10735405

[CR32] Fitzpatrick, P., Romero, V., Amaral, J. L., Duncan, A., Barnard, H., Richardson, M. J., & Schmidt, R. C. (2017). Evaluating the importance of social motor synchronization and motor skill for understanding autism. *Autism Research*, *10*(10), 1687–1699. 10.1002/aur.1808. 28590041 10.1002/aur.1808 PMC5648610

[CR33] Flach, R., Knoblich, G., & Prinz, W. (2004). The two-thirds power law in motion perception. *Visual Cognition*, *11*(4), 461–481. 10.1080/13506280344000392.

[CR34] Focaroli, V., Taffoni, F., Parsons, S. M., Keller, F., & Iverson, J. M. (2016). Performance of motor sequences in children at heightened vs. low risk for ASD: A longitudinal study from 18 to 36 months of age. *Frontiers in Psychology*, *7*(MAY), 1–9. 10.3389/fpsyg.2016.00724. 27242630 10.3389/fpsyg.2016.00724 PMC4865480

[CR35] Forti, S., Valli, A., Perego, P., Nobile, M., Crippa, A., & Molteni, M. (2011). Motor planning and control in autism. A kinematic analysis of preschool children. *Research in Autism Spectrum Disorders*, *5*(2), 834–842. 10.1016/J.RASD.2010.09.013.

[CR37] Fourie, E., Palser, E. R., Pokorny, J. J., Neff, M., & Rivera, S. M. (2020). Neural Processing and production of gesture in children and adolescents with Autism Spectrum Disorder. *Frontiers in Psychology*, *10*, 10.3389/fpsyg.2019.03045. 10.3389/fpsyg.2019.03045 PMC6987472 32038408

[CR38] Gallese, V., Rochat, M. J., & Berchio, C. (2013). The mirror mechanism and its potential role in autism spectrum disorder. *Developmental Medicine and Child Neurology*, *55*(1), 15–22. 10.1111/j.1469-8749.2012.04398.x. 22924341 10.1111/j.1469-8749.2012.04398.x

[CR39] Glazebrook, C. M., Elliott, D., & Lyons, J. (2016). A Kinematic Analysis of How Young Adults with and Without Autism Plan and Control Goal-Directed Movements. In *Motor Control* (Vol. 10, Issue 3). 10.1123/mcj.10.3.244. 10.1123/mcj.10.3.244 17106133

[CR40] Gordon, R. G., & Watson, L. R. (2015). Brief report: Gestures in children at risk for Autism Spectrum disorders. *Journal of Autism and Developmental Disorders*, *45*(7), 2267–2273. 10.1007/s10803-015-2390-0. 25676685 10.1007/s10803-015-2390-0

[CR41] Grace, N., Enticott, P. G., Johnson, B. P., & Rinehart, N. J. (2017). Do handwriting difficulties correlate with Core Symptomology, Motor proficiency and attentional behaviours? *Journal of Autism and Developmental Disorders*, *47*(4), 1006–1017. 10.1007/s10803-016-3019-7. 28083779 10.1007/s10803-016-3019-7

[CR42] Harrison, L. A., Kats, A., Kilroy, E., Butera, C., Jayashankar, A., Keles, U., & Aziz-Zadeh, L. (2021). Motor and sensory features successfully decode autism spectrum disorder and combine with the original RDoC framework to boost diagnostic classification. *Scientific Reports*, *11*(1). 10.1038/s41598-021-87455-w. 10.1038/s41598-021-87455-w PMC8035204 33837251

[CR43] Hicheur, H., Vieilledent, S., Richardson, M. J. E., Flash, T., & Berthoz, A. (2005). Velocity and curvature in human locomotion along complex curved paths: A comparison with hand movements. *Experimental Brain Research*, *162*(2), 145–154. 10.1007/s00221-004-2122-8. 15586276 10.1007/s00221-004-2122-8

[CR44] Holloway, J. M., Tomlinson, S. M., & Hardwick, D. D. (2022). Strategies to support learning of Gross Motor tasks in children with Autism Spectrum disorder: A scoping review. *Physical & Occupational Therapy in Pediatrics*, 1–17. 10.1080/01942638.2022.2073800. 10.1080/01942638.2022.2073800 35538730

[CR45] Ivanenko, Y. P., Grasso, R., Macellari, V., & Lacquaniti, F. (2002). Control of foot trajectory in human locomotion: Role of ground contact forces in simulated reduced gravity. *Journal of Neurophysiology*, *87*(6), 3070–3089. 10.1152/jn.2002.87.6.3070. 12037209 10.1152/jn.2002.87.6.3070

[CR46] Iverson, J. M. (2010). Developing language in a developing body: The relationship between motor development and language development. *Journal of Child Language*, *37*(2), 229–261. 10.1017/S0305000909990432. 20096145 10.1017/S0305000909990432 PMC2833284

[CR47] Iverson, J. M., Shic, F., Wall, C. A., Chawarska, K., Curtin, S., Estes, A., Gardner, J. M., Hutman, T., Landa, R. J., Levin, A. R., Libertus, K., Messinger, D. S., Nelson, C. A., Ozonoff, S., Sacrey, L. A. R., Sheperd, K., Stone, W. L., Tager-Flusberg, H. B., Wolff, J. J., & Young, G. S. (2019). Early motor abilities in infants at heightened versus low risk for ASD: A Baby Siblings Research Consortium (BSRC) study. *Journal of Abnormal Psychology*, *128*(1), 69–80. 10.1037/abn0000390. 30628809 10.1037/abn0000390 PMC6338079

[CR48] Kandel, S., Orliaguet, J. P., & Viviani, P. (2000). Perceptual anticipation in handwriting: The role of implicit motor competence. *Perception and Psychophysics*, *62*(4), 706–716. 10.3758/BF03206917. 10883579 10.3758/bf03206917

[CR49] LeBarton, E. S., & Landa, R. J. (2019). Infant motor skill predicts later expressive language and autism spectrum disorder diagnosis. *Infant Behavior and Development*, *54*, 37–47. 10.1016/j.infbeh.2018.11.003. 30557704 10.1016/j.infbeh.2018.11.003

[CR50] Levit-Binnun, N., Schechtman, E., & Flash, T. (2006). On the similarities between the perception and production of elliptical trajectories. *Experimental Brain Research*, *172*(4), 533–555. 10.1007/s00221-006-0355-4. 16501963 10.1007/s00221-006-0355-4

[CR51] Licari, M. K., Varcin, K., Hudry, K., Leonard, H. C., Alvares, G. A., Pillar, S. V., Stevenson, P. G., Cooper, M. N., & Whitehouse, A. J. O. (2021). The course and prognostic capability of motor difficulties in infants showing early signs of autism. *Autism Research*, *aur.2545*, 10.1002/aur.2545. 10.1002/aur.2545 34021977

[CR52] Lu, S. C., Rowe, P., Tachtatzis, C., Andonovic, I., Anzulewicz, A., Sobota, K., & Delafield-Butt, J. (2022). Swipe kinematic differences in young children with autism spectrum disorders are task- and age-dependent: A smart tablet game approach. *Brain Disorders*, *5*, 100032. 10.1016/J.DSCB.2022.100032.

[CR53] Méary, D., Kitromilides, E., Mazens, K., Graff, C., & Gentaz, E. (2007). Four-day-old human neonates look longer at non-biological motions of a single point-of-light. *Plos One*, *2*(1), e186. 10.1371/journal.pone.0000186. 17264887 10.1371/journal.pone.0000186 PMC1779622

[CR54] Meirovitch, Y., Harris, H., Dayan, E., Arieli, A., & Flash, T. (2015). Alpha and beta band event-related desynchronization reflects kinematic regularities. *Journal of Neuroscience*, *35*(4), 1627–1637. 10.1523/JNEUROSCI.5371-13.2015. 25632138 10.1523/JNEUROSCI.5371-13.2015 PMC6795263

[CR55] Millar, L., McConnachie, A., Minnis, H., Wilson, P., Thompson, L., Anzulewicz, A., Sobota, K., Rowe, P., Gillberg, C., & Delafield-Butt, J. (2019). Phase 3 diagnostic evaluation of a smart tablet serious game to identify autism in 760 children 3–5 years old in Sweden and the United Kingdom. *British Medical Journal Open*, *9*(7), 1–7. 10.1136/bmjopen-2018-026226. 10.1136/bmjopen-2018-026226 PMC6661582 31315858

[CR57] Mosconi, M. W., & Sweeney, J. A. (2015). Sensorimotor dysfunctions as primary features of autism spectrum disorders. *Science China Life Sciences*, *58*(10), 1016–1023. 10.1007/S11427-015-4894-4. 26335740 10.1007/s11427-015-4894-4 PMC5304941

[CR56] Mosconi, M. W., Mohanty, S., Greene, R. K., Cook, E. H., Vaillancourt, D. E., & Sweeney, J. A. (2015). Feedforward and Feedback Motor Control Abnormalities Implicate Cerebellar Dysfunctions in Autism Spectrum Disorder. *Journal of Neuroscience*, *35*(5), 2015–2025. 10.1523/JNEUROSCI.2731-14.2015. 25653359 10.1523/JNEUROSCI.2731-14.2015 PMC4315832

[CR58] Nobile, M., Perego, P., Piccinini, L., Mani, E., Rossi, A., Bellina, M., & Molteni, M. (2011). Further evidence of complex motor dysfunction in drug naïve children with autism using automatic motion analysis of gait. *SAGE Publications and the National Autistic Society*, *15*(3), 1362–3613. 10.1177/1362361309356929. 10.1177/1362361309356929 21478224

[CR59] Patterson, J. W., Armstrong, V., Duku, E., Richard, A., Franchini, M., Brian, J., Zwaigenbaum, L., Bryson, S. E., Sacrey, L. A. R., Roncadin, C., & Smith, I. M. (2021). Early trajectories of motor skills in infant siblings of children with autism spectrum disorder. *Autism Research*. 10.1002/AUR.2641. 34826349 10.1002/aur.2641

[CR60] Richardson, M. J. E., & Flash, T. (2002). Comparing smooth arm movements with the two-thirds power law and the related segmented-control hypothesis. *The Journal of Neuroscience: The Official Journal of the Society for Neuroscience*, *22*(18), 8201–8211. 10.1523/JNEUROSCI.22-18-08201.2002. 12223574 10.1523/JNEUROSCI.22-18-08201.2002 PMC6758108

[CR61] Rinehart, N. J., Tonge, B. J., Bradshaw, J. L., Iansek, R., Enticott, P. G., & McGinley, J. (2006a). Gait function in high-functioning autism and Asperger’s disorder: Evidence for basal-ganglia and cerebellar involvement? *European Child and Adolescent Psychiatry*, *15*(5), 256–264. 10.1007/s00787-006-0530-y. 16554961 10.1007/s00787-006-0530-y

[CR62] Rinehart, N. J., Tonge, B. J., Iansek, R., McGinley, J., Brereton, A. V., Enticott, P. G., & Bradshaw, J. L. (2006b). Gait function in newly diagnosed children with autism: Cerebellar and basal ganglia related motor disorder. *Developmental Medicine and Child Neurology*, *48*(10), 819–824. 10.1017/S0012162206001769. 16978461 10.1017/S0012162206001769

[CR63] Roid, G. H., & Pomplun, M. (2012). The Stanford-Binet Intelligence scales, Fifth Edition. *Contemporary intellectual assessment: Theories, tests, and issues* (3rd ed., pp. 249–268). The Guilford Press.

[CR64] Salomon, R., Goldstein, A., Vuillaume, L., Faivre, N., Hassin, R. R., & Blanke, O. (2016). Enhanced discriminability for nonbiological motion violating the two-thirds power law. *Journal of Vision*, *16*(8), 12. 10.1167/16.8.12. 27299772 10.1167/16.8.12

[CR65] Schwartz, A. B., & Moran, D. W. (1999). Motor cortical activity during drawing movements: Population representation during lemniscate tracing. *Journal of Neurophysiology*, *82*(5), 2705–2718. 10.1152/jn.1999.82.5.2705. 10561439 10.1152/jn.1999.82.5.2705

[CR66] Schwartz, A. B., & Moran, D. W. (2000). Arm trajectory and representation of movement processing in motor cortical activity. *European Journal of Neuroscience*, *12*, 1851–1856. https://pdfs.semanticscholar.org/54fd/0e0f4bb157a47a8fc37676a8a1ce53cc8f68.pdf. 10.1046/j.1460-9568.2000.00097.x 10886326

[CR67] Sciaky, R., Lacquaniti, F., Terzuolo, C., & Soechting, J. F. (1987). A note on the kinematics of drawing movements in children. *Journal of Motor Behavior*, *19*(4), 518–525. 10.1080/00222895.1987.10735427. 15136275 10.1080/00222895.1987.10735427

[CR68] Sparrow, S. S., Cicchetti, D., & Balla, D. A. (2005). *Vineland Adaptive Behavior Scales, Second Edition (Vineland-II)*.

[CR69] Staples, K. L., & Reid, G. (2010). Fundamental Movement Skills and Autism Spectrum Disorders. *Journal of Autism and Developmental Disorders*, *40*(2), 209–217. 10.1007/s10803-009-0854-9. 19685284 10.1007/s10803-009-0854-9

[CR70] Todorov, E., & Jordan, M. I. (1998). Smoothness maximization along a predefined path accurately predicts the speed profiles of Complex Arm movements. *Journal of Neurophysiology*, *80*(2), 696–714. 10.1152/jn.1998.80.2.696. 9705462 10.1152/jn.1998.80.2.696

[CR71] Torres, E. B., Brincker, M., Isenhower, R. W., Yanovich, P., Stigler, K., Nurnberger, J., Metaxas, D. N., & José, J. V. (2013). Autism: The Micro-Movement perspective. *Frontiers in Integrative Neuroscience*, *0*(July 2013), 32. 10.3389/FNINT.2013.00032/BIBTEX. 10.3389/fnint.2013.00032 PMC3721360 23898241

[CR72] Travers, B. G., Bigler, E. D., Tromp, D. P. M., Adluru, N., Destiche, D., Samsin, D., Froehlich, A., Prigge, M. D. B., Duffield, T. C., Lange, N., Alexander, A. L., & Lainhart, J. E. (2015). Brainstem White Matter Predicts Individual Differences in Manual Motor Difficulties and Symptom Severity in Autism. *Journal of Autism and Developmental Disorders*, *45*(9), 3030–3040. 10.1007/s10803-015-2467-9. 26001365 10.1007/s10803-015-2467-9 PMC4554823

[CR74] Trevarthen, C., & Delafield-Butt, J. T. (2013). Autism as a developmental disorder in intentional movement and affective engagement. *Frontiers in Integrative Neuroscience*, *7*(49), 1–16. 10.3389/fnint.2013.00049. 23882192 10.3389/fnint.2013.00049 PMC3713342

[CR73] Trevarthen, C., & Delafield-Butt, J. (2017). Development of consciousness. *The Cambridge Encyclopedia of Child Development*, 821–835. 10.1017/9781316216491.131.

[CR75] Viviani, P., & Flash, T. (1995). Minimum-Jerk, Two-Thirds Power Law, and Isochrony: Converging Approaches to Movement Planning. In *Journal of Experimental Psychology: Human Perception and Performance* (Vol. 21, Issue 1). https://pdfs.semanticscholar.org/ea7e/c6fa77e403cdc0a9b5575a7e32fd6cc79a07.pdf. 10.1037//0096-1523.21.1.32 7707032

[CR76] Viviani, P., & Schneider, R. (1991). A Developmental Study of the Relationship Between Geometry and Kinematics in Drawing Movements. In *Journal of Experimental Psychology: Human Perception and Performance* (Vol. 17, Issue 1). https://pdfs.semanticscholar.org/e296/71b68640b7594cc8cd626d65e26ee5148612.pdf?_ga=2.146849073.727053310.1555449274-1885823519.1554937831. 10.1037//0096-1523.17.1.198 1826312

[CR77] Viviani, P., & Stucchi, N. (1992). Biological Movements look Uniform: Evidence of motor-perceptual interactions. *Journal of Experimental Psychology: Human Perception and Performance*, *18*(3), 603–623. 10.1037/0096-1523.18.3.603. 1500865 10.1037//0096-1523.18.3.603

[CR78] Von Hofsten, C. (2007). Action in development. *Developmental Science*, *10*(1), 54–60. 10.1111/j.1467-7687.2007.00564.x. 17181700 10.1111/j.1467-7687.2007.00564.x

[CR79] von Hofsten, C., & Rönnqvist, L. (1993). The structuring of neonatal arm movements. *Child Development*, *64*(4), 1046–1057. 10.1111/J.1467-8624.1993.TB04187.X. 8404256

[CR80] Wang, Z., Magnon, G. C., White, S. P., Greene, R. K., Vaillancourt, D. E., & Mosconi, M. W. (2015). Individuals with autism spectrum disorder show abnormalities during initial and subsequent phases of precision gripping. *Journal of Neurophysiology*, *113*(7), 1989–2001. 10.1152/JN.00661.2014/ASSET/IMAGES/LARGE/Z9K0061529150007.JPEG. 25552638 10.1152/jn.00661.2014 PMC4416549

[CR81] Wann, J., Nimmo-Smith, I., & Wing, A. M. (1988). Relation between velocity and curvature in Movement: Equivalence and divergence between a Power Law and a minimum-jerk model. *Journal of Experimental Psychology: Human Perception and Performance*, *14*(4), 622–637. 10.1037/0096-1523.14.4.622. 2974873 10.1037//0096-1523.14.4.622

[CR82] Wechsler, D. (2011). *Wechsler Abbreviated Scale of Intelligence, Second Edition (WASI-II)*. NCS Pearson.

[CR83] Whyatt, C., & Craig, C. M. (2013). Interceptive skills in children aged 9–11 years, diagnosed with Autism Spectrum Disorder. *Research in Autism Spectrum Disorders*, *7*(5), 613–623. 10.1016/J.RASD.2013.01.003. 10.1007/s10803-011-1421-8 22180003

[CR84] Williams, J. H. G., Whiten, A., & Singh, T. (2004). A systematic review of Action Imitation in Autistic Spectrum Disorder. *Journal of Autism and Developmental Disorders*, *34*(3), 285–299. 15264497 10.1023/b:jadd.0000029551.56735.3a

[CR85] Wilson, R. B., Vangala, S., Elashoff, D., Safari, T., & Smith, B. A. (2021). Using Wearable Sensor Technology to measure motion complexity in infants at high familial risk for Autism Spectrum Disorder. *Sensors (Basel Switzerland)*, *21*(2), 1–13. 10.3390/S21020616. 10.3390/s21020616 PMC7830886 33477359

[CR86] Yang, H. C., Lee, I. C., & Lee, I. C. (2014). Visual feedback and target size effects on Reach-to-grasp tasks in children with autism. *Journal of Autism and Developmental Disorders*, *44*(12), 3129–3139. 10.1007/s10803-014-2165-z. 24974254 10.1007/s10803-014-2165-z

[CR87] Yang, Y. C., Lu, L., Jeng, S. F., Tsao, P. N., Cheong, P. L., Li, Y. J., Wang, S. Y., Huang, H. C., & Wu, Y. T. (2019). Multidimensional Developments and Free-Play Movement Tracking in 30- to 36-Month-Old toddlers with Autism Spectrum Disorder who were full term. *Physical Therapy*, *99*(11), 1535–1550. 10.1093/ptj/pzz114. 31392998 10.1093/ptj/pzz114

[CR88] Zhao, Z., Tang, H., Alviar, C., Christopher, |, Kello, T., Zhang, Xiaobin, Hu, X., Qu, X., & Lu, J. (2021). Excessive and less complex body movement in children with autism during face-to-face conversation: An objective approach to behavioral quantification. *Autism Research*. 10.1002/AUR.2646. 34837352 10.1002/aur.2646

